# Resonant plasma excitation by single-cycle THz pulses

**DOI:** 10.1038/s41598-017-18312-y

**Published:** 2018-01-18

**Authors:** A. Curcio, A. Marocchino, V. Dolci, S. Lupi, M. Petrarca

**Affiliations:** 1 Department of Basic and Applied Sciences for Engineering (SBAI), “Sapienza” University of Rome, Via A. Scarpa 14, 00161 Rome, Italy; 2 Roma1-INFN, P.le Aldo Moro, 2, 00185 Rome, Italy; 3 INFN-LNF, via Enrico Fermi 40, 00044 Frascati, Italy; 4 Department of Physics, “Sapienza” University of Rome, Piazzale A. Moro 2, I-00185 Rome, Italy

**Keywords:** Applied optics, Terahertz optics

## Abstract

In this paper, an alternative perspective for the generation of millimetric high-gradient resonant plasma waves is discussed. This method is based on the plasma-wave excitation by energetic single-cycle THz pulses whose temporal length is comparable to the plasma wavelength. The excitation regime discussed in this paper is the quasi-nonlinear regime that can be achieved when the normalized vector potential of the driving THz pulse is on the order of unity. To investigate this regime and determine the strength of the excited electric fields, a Particle-In-Cell (PIC) code has been used. It has been found that by exploiting THz pulses with characteristics currently available in laboratory, longitudinal electron plasma waves with electric gradients up to hundreds MV/m can be obtained. The mm-size nature of the resonant plasma wave can be of great utility for an acceleration scheme in which high-brightness electron bunches are injected into the wave to undergo a strong acceleration. The long-size nature of the acceleration bucket with respect to the short length of the electron bunches can be handled in a more robust manner in comparison with the case when micrometric waves are employed.

## Introduction

Terahertz radiation (1 THz corresponds to ∼4 meV photon energy, or ∼300 *μ*m radiation wavelength) has a strong impact in many areas of research, spanning the quantum control of materials^1–4^, plasmonics^5–8^, and tunable optical devices based on Dirac-electron systems^9^ to technological applications such as medical imaging and security^3,10^. Recently, striking applications for novel acceleration techniques exploiting high-intensity THz radiation have also been proposed and successfully tested^11–14^. Despite the strong interest triggered by these results, experimental work remains at an early stage, being not yet competitive with conventional RF-based acceleration schemes. In the previous works^11–13^, proof of principle electron acceleration experiments induced by THz pulses have been reported, therein showing that a strong THz field can be used to boost the electron energy in a short space interval. In these works, the net energy gain imposed on the non-relativistic electrons was on the order of a few keV. Moreover, the acceleration scheme relied on the interaction of the electrons with a THz field contained in dedicated waveguide structures under vacuum conditions.

In this paper, the possibility of using a single-cycle THz pulse to efficiently excite resonant mm-size electron plasma waves will be discussed. This interaction is expected to produce high-gradient longitudinal electric wakefields in the some tens of MV/m range and higher. In particular, it is proposed to exploit these wakefields in the LINAC-type acceleration scheme in which pre-existing electron bunches are injected in the accelerating wave to undergo a strong acceleration. This scheme enriches the field concerning acceleration techniques based on plasma waves.

THz pulses can be generated in different ways, and extensive reviews can be found in^2–4^. Two main techniques can be identified: accelerator-and laser-based techniques. In the former, THz pulses are generated by transition or diffraction radiation triggered by the field of electron bunches interacting with thin foils (metal or dielectric). The main limitation of this technique resides in the large size (tens of meters) of the LINAC required to produce THz pulses in the tens of microjoules energy range^15,16^. For laser-triggered THz emission, the major processes commonly used are the photoconductive switching^3^, Optical Rectification (OR) and air-plasma techniques. In particular, in OR^2–4^ a high-power near-infrared laser (pump laser) is transduced through non-linear optical crystals, such as ZnTe, LiNbO_3_, and GaAs crystals, into a THz pulse. In contrast, the air-plasma scheme^3,17–25^, is based on the possibility of focusing in air a femtosecond laser pulse at the fundamental frequency *ω* together with its second harmonic at 2*ω*. The resulting pulse undergoes filamentation, creating cm-long plasmas via multiphoton/tunneling ionization, and energetic THz pulses are emitted in the forward direction. The generation mechanism has been described in terms of the photocurrent quantum mechanical model^21–23^, in which the THz signal arises from a non-vanishing transverse plasma current appearing when the ionization is triggered by an asymmetric laser field, as for example the field originated by *ω* − 2*ω* field superposition with a proper relative phase. Recently, this model was further investigated and refined in^25^. The main limitation of the OR technique is related to a low conversion efficiency, which is, in most of the inorganic non-linear crystals previously cited, on the order of 0.1–0.2 %. Recently, a strong increase in the conversion efficiency was achieved through the use of organic non-linear crystals, such as DAST, DMST and OH1, which show a 2–3% efficiency when illuminated by a Cr-Forsterite fs high-power laser emitting at approximately 1240 nm. In this case, a single-cycle THz pulse centered around λ_*THz*_ ≃ 100 *μ*m has been obtained, having an energy/pulse of ≃1 mJ^26,27^. Moreover, the possibility of producing larger organic single crystals, and/or a mosaic structure^28^ to increase the crystal surface, has been suggested to be a viable path to exploiting higher energy pump lasers, thereby producing higher energy (>1 mJ) THz pulses with higher intensity and electric field. The air-plasma scheme is a promising method for the generation of high-intensity THz pulses. To date, an energy level of ∼100 *μ*J has been produced, and the development of mid-infrared (2–6 *μ*m) high-energy (tens of mJ) short-pulse (50 fs) laser systems^29^ together with a favorable scaling law^30^ for generation efficiency of the air-plasma technique could make this technique very competitive with the OR technique. As discussed above, a strong worldwide effort in the scientific community to find amplification schemes for THz radiation is already ongoing, thereby increasing the probability of obtaining more energetic THz pulses in the near future^31,32^.

In this regard, we chose as a starting point for the discussion presented in this paper a THz pulse with similar characteristics to^26,27^, and reported in what follows. The main reason for this resides in the fact that a THz pulse energy of ≃1 mJ can already generate a wakefield in the MV/m range in the quasi-nonlinear regime, as will be demonstrated through this work; therefore, this THz energy value can already be of practical interest.

## Motivations

The potentiality of THz pulses for unconventional acceleration schemes resides in the long wavelength nature of the THz radiation (λ_*THz*_ ≃ 100 *μ*m) in comparison with infrared or near-infrared radiation such as CO_2_ (10.6 *μ*m) or Ti:Sa (0.8 *μ*m) laser pulses. Moreover, from Maxwell’s equations, the non-linear term driving the electron plasma waves, related to the ponderomotive force^33–37^, is proportional at low driver intensities to the gradient of the squared vector potential *F*_*pond*_ ∝ ∇*A*^2^, which has a normalized peak amplitude expressed by the relation ${{\rm{a}}}_{0}=8.5\times {10}^{-10}\,{\rm{\lambda }}[{\mu }m]\sqrt{{I}[{W}/{c}{{m}}^{2}]}$. Therefore, for a fixed pulse intensity, the amplitude of the electron plasma wave scales proportionally to λ^2^. In the so-called linear regime, a_0_ ≪ 1, where a_0_ is the normalized quiver momentum of the electrons moving under the action of the vector potential. Single-cycle THz pulses, as produced in^26^, are suitable to efficiently excite mm-size plasma wakefields. This is because the electron plasma wave excitation on the wake of an electromagnetic pulse is a resonant mechanism related to the matching of the driver pulse duration L to the plasma wavelength λ_*p*_ = 2π*c*/*ω*_*p*_, where ${{\omega }}_{p}=\sqrt{{n}_{0}{e}^{2}/{{\varepsilon }}_{0}m}$^33^ is the proper frequency for collective plasma electron oscillations. The background electron plasma density has been denoted by *n*_0_, the elementary charge and the electron mass as *e* and *m* respectively, and the vacuum dielectric constant as ε_0_. The condition of perfect matching corresponds to the best combination of values of the electron plasma density n_0_ and driver pulse length L that maximizes the peak amplitude of the generated electron plasma wave. In particular, in the linear theory of the laser-driven plasma excitation for the wakefield acceleration scheme, the amplitude of the generated wakefield depends on the ratio ${\rm{L}}/{{\rm{\lambda }}}_{p}\propto \sqrt{{n}_{0}}$L, and it varies for different laser pulse shapes. For a cos-like driver pulse such as the pulse used in the next section for the PIC simulations, a detailed study has already been performed^33^, therein demonstrating that the perfect ratio is ${\rm{L}}/{{\rm{\lambda }}}_{p}\sim \mathrm{1/2}$.

In other words the ponderomotive force acts like a pressure term displacing the plasma electrons from the high-intensity region of the driver pulse and when the driver pulse length is comparable with the electron plasma wavelength, the plasma resonance is excited with high efficiency, slightly similar to what occurs to a harmonic oscillator when an external oscillating force is imposed at the proper frequency of the system.

As stated at the beginning of this work, we are interested in the generation of a plasma wakefield with λ_*p*_ ≃ 1 mm (corresponding to a period of ≃3 ps). Therefore, by exploiting single-cycle THz pulses (as in^26,27^,) with central frequency *v*_0_ ≃ 3 THz, the value of L ≃ 105 *μ*m ≃ λ_*p*_/10 is a fraction of the plasma wavelength, resulting in a factor of 5 out of resonance. Nevertheless, this operating point can be considered as “reasonable” because it yields wakefield values already in the MV/m range; this is a range that is already interesting for applications.

It is important to remark that this configuration of parameters may be advantageous for plasma-based accelerators. The external injection of high-brightness electron bunches in millimetric electron plasma waves excited by low-intensity THz pulses (*a*_0_ < 1) can be utilized as an acceleration scheme, being stable in terms of energy fluctuations of the accelerated electron bunches and convenient in terms of synchronization and energy-spread restraints. The main cause resides in the much greater length λ_*p*_/4 of the accelerating and focusing bucket with respect to the length *L*_*e*_ ≃ 50 *μ*m of high-brightness electron bunches that are currently commonly producible^38,39^. It is well known^40^ that under this condition (*L*_*e*_ << λ_*p*_/4), all the electrons in the bunch will experience the same longitudinal accelerating field, thereby favoring the preservation of the small initial energy spread during the acceleration. Moreover, the required synchronization and relative jitter between the electron bunch and the accelerating field can be estimated to be on the order of ≃100 fs, i.e., much less stringent than the 10 fs or less required if higher density plasmas (10^17−18^ cm^−3^ corresponding to ${{\rm{\lambda }}}_{p}\simeq 100-34\,\mu$m) are employed^40^. Nevertheless, these aspects will be investigated in a more detailed and dedicated work attempting to provide insights into the acceleration process.

For all the reasons discussed above, THz-pulse-driven wakefield excitation can be an interesting scheme for strongly accelerating high-brightness low-energy-spread electron bunches within compact electron facilities that are of particular interest, e.g., for medical applications^41–44^.

## Simulations and Discussion

**Figure 1:**
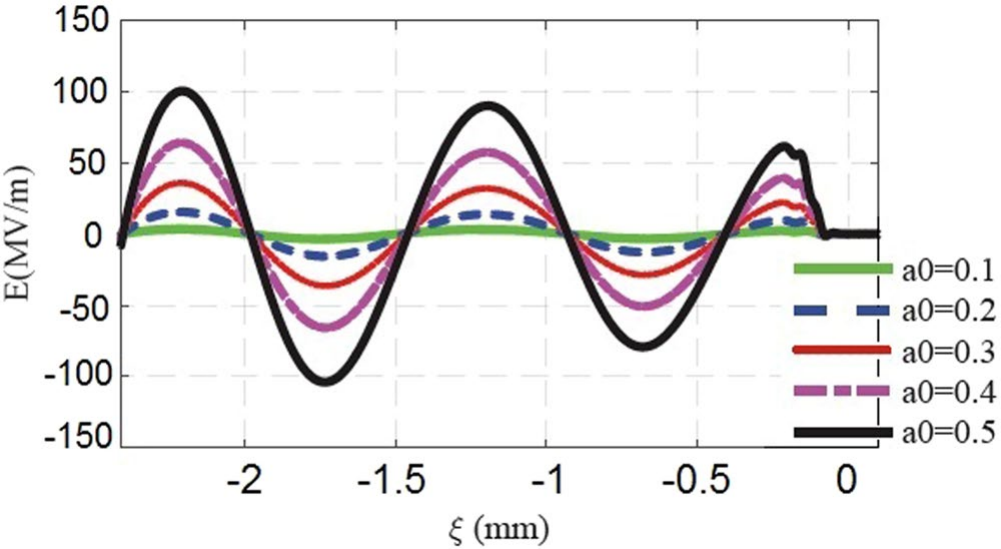
Axial electric field *E* driven by a THz pulse located in the region −2 L < ξ < 0 (the pulse is moving to the right) for different values of a_0_, where λ_*p*_ = 1 mm (n_0_ = 1 × 10^15^ cm^−3^ plasma density), L = 105 *μ*m.

**Figure 2:**
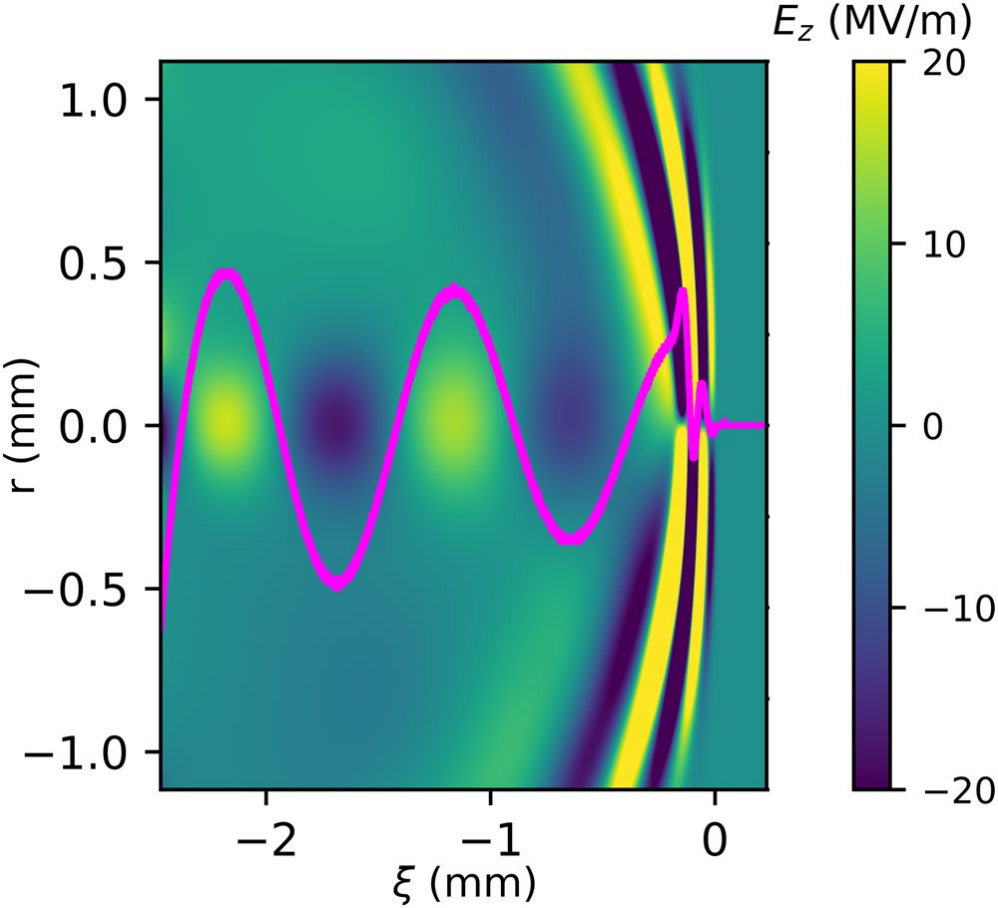
2D map of the total longitudinal electric field for the input parameters a_0_ = 0.2, λ_*p*_ = 1 mm (n_0_ = 1 × 10^15^ cm^−3^), and L = 105 *μ*m. Starting from right: fast field component oscillating at twice the frequency of the THz driver followed by the generated electron plasma wakefield after 2.5 mm of propagation inside the plasma. The axial wakefield lineout is also shown in violet, with a peak amplitude of 16 MV/m for a_0_ = 0.2.

To study and validate the plasma response dynamics for different values of the THz pulse intensity and to estimate the values of the peak accelerating fields, we have performed Particle-in-Cell (PIC) simulations with the ALaDyn code^45–47^. The 3D parallel PIC code has been developed and optimized for Laser WakeField Acceleration (LWFA) as well as plasma wakefield acceleration scenarios. ALaDyn is currently used to support different experimental campaigns concerning the interaction of high-intensity lasers with plasma^48,49^. For our studies, we run simulations in slab bi-dimensional simmetry. These simulations are required to explore a new regime characterized by a single-cycle THz pulse propagating in an underdense plasma with a_0_ values in the quasi-nonlinear range 0.1 < a_0_ <1. The propagation distance of 2.5 mm in the initially homogeneous electron plasma density has been chosen to optimize the computational cost of each simulation while simultaneously avoiding artifacts from numerical reflection of the THz field reaching the margin of the transverse size of the numerical box while diffracting. Simulations have been performed with a longitudinal cell resolution of 0.2 *μ*m and a transverse resolution of 1.6 *μ*m. The mesh, to accommodate multiple oscillations, is set to be equal to 51000 points longitudinally and 1440 transversally. The final plot, to avoid numerical noise, is run with 25 particles per cell. The normalized vector potential adopted in the simulation is

1 $$a(r,z,t)={a}_{0}\,\exp (-{r}^{2}/{w}_{0}^{2})\,{\cos }^{2}({\rm{\pi }}{\rm{\xi }}\mathrm{/2}L)\,\cos (2{\rm{\pi }}{\rm{\xi }}/{{\rm{\lambda }}}_{THz}).$$

for −2*L* < *ξ* < 0 and *a*(*r*, *z*, *t*) = 0 otherwise. This represents a linearly polarized single-cycle THz pulse whereby *L* = 105 *μ*m and w_0_ = 270 *μ*m are the longitudinal and radial pulse dimensions, respectively^26,27^, and *ξ* = *z* − *ct* is the co-moving coordinate. Results for the on-axis wakefields assuming an initial plasma density of n_0_ = 1 × 10^15^ cm^−3^ are shown in Fig. 1 for different values of a_0_. For a given a_0_, the wakefield behavior shown in Fig. 1 presents a quasi-sinusoidal shape with a decreasing amplitude due to the decrease in the intensity of the THz pulse caused by diffraction. The wakefield wavelength is ∼λ_*p*_, which is a characteristic of the quasi-nonlinear regime^33^. Figure 2 shows a 2D map of the total longitudinal electric field of the system (made by the THz pulse and the plasma) for the input parameters a_0_ = 0.2, λ_*p*_ = 1 mm (n_0_ = 1 × 10^15^ cm^−3^), L = 105 *μ*m and for ∼2.5 mm propagation length of the driver inside the plasma. It has been decided to show the 2D structure of the expected plasma wakefield for a_0_ = 0.2 because this input value refers to a THz pulse that can be produced currently in laboratory following the method reported in^26^. The mm-size plasma wakefield component covers the region −2.5 mm < *ξ* < −1 mm behind the THz pulse, and it is the component we are interested in for the acceleration of externally injected electron bunches. On the right, a fast electric field component oscillating at twice the driver frequency is shown in correspondence of the longitudinal position of the driver pulse. This component is the effect of the driver-plasma local interaction^33^. In Fig. 2, the purple line is a lineout of the axial (r = 0) total longitudinal electric field showing a peak amplitude of 16 MV/m for a_0_ = 0.2. In the external injection scheme, the electron bunches to be accelerated have a transverse beam dimension smaller than 100 *μm*. In fact if the electron bunch transverse dimension is much smaller than the THz pulse transverse extension, then the electrons will experience nearly the same wakefield with an amplitude value closer to the axial electric field one (see Fig. 2). Thus, the axial electric field gives a reasonable estimation of the accelerating field which can be exploited in such experiments. Usually the transverse wakefields (not shown) can be as strong as the longitudinal ones, nevertheless they affect the electron acceleration only in the sense that they provide (de)focusing during the acceleration^33^. Nevertheless the study of the beam transverse dynamics is beyond the purpose of the current paper.

Moreover, we have found that the equation known to be valid for the 1D non-linear regime and for a linearly polarized flat-top pulse profile with longitudinal length matched to the plasma wavelength^33^

2 $${E}_{N}(V/m)={E}_{wb}\frac{{a}_{0}^{2}}{2\sqrt{1+\frac{{a}_{0}^{2}}{2}}},$$

where ${E}_{wb}=m{{\omega }}_{p}c/e\sim 96\sqrt{{n}_{0}(c{m}^{-3})}[V/m]$, can be used as a reference for the scaling behavior of the wakefield peak amplitude. Under this assumption, a discrepancy on the percent level is found with respect to the wakefield peak amplitude obtained from the 2D PIC simulations. It is important to note that since Eq. (2) is retrieved under the matching condition assumption^33^, it yields the maximum field amplitude obtainable for that specific driver pulse.

**Figure 3:**
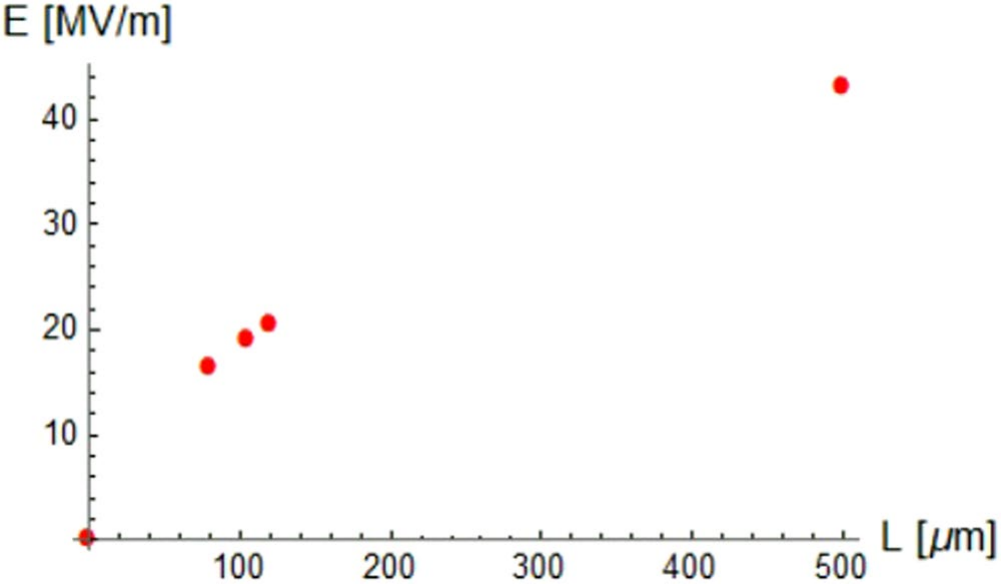
Peak electric field versus the THz pulse length for a fixed background electron plasma density of n_0_ = 10^15^ cm^−3^. The red points represent the peak value of the wakefield simulated with the PIC code for different choices of the THz pulse length driving the electron plasma wave.

Because the THz pulse length can be experimentally controlled with high accuracy (deviation of a few percent) by standard techniques, the plasma-wakefield excitation has been investigated for two other values of the pulse length L (80 *μ*m and 120 *μ*m at a fixed value of n_0_ = 10^15^ cm^−3^) to supply an upper and lower bound for the wakefield amplitude generated by a driver pulse exciting a plasma density of 10^15^ *cm*^−3^ and with a pulse length varying ∼20% from the nominal one; see Fig. 3. Moreover, in the same Fig. 3, the highest field amplitude that can be obtained for a_0_ = 0.2 under the matching condition ($L/{{\rm{\lambda }}}_{p}\sim \mathrm{1/2}$) is also shown for completeness.

**Figure 4:**
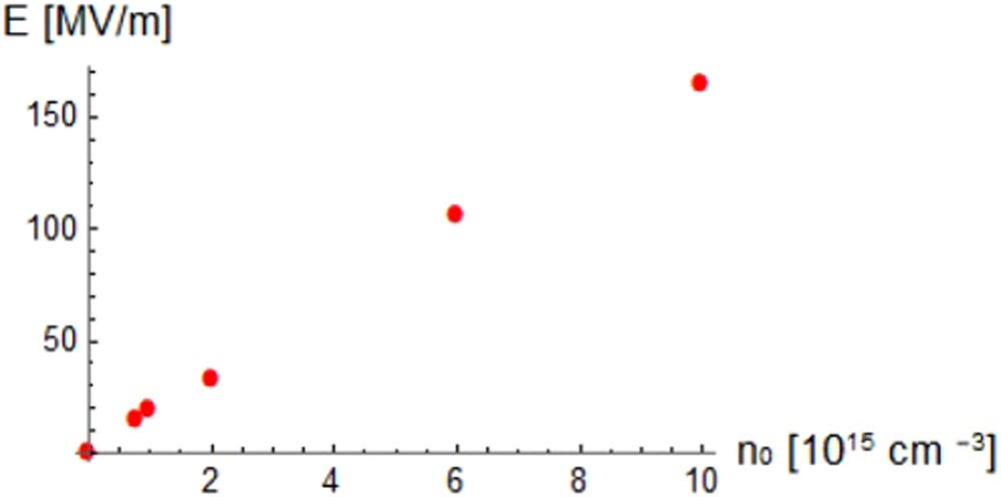
Peak electric field versus the background electron plasma density for a fixed THz pulse length of L = 105 *μ*m. The points represent the peak value of the wakefield simulated with the PIC code for different choices of initial background electron plasma density.

The plasma density can be generated with a ten percent deviation from the nominal density chosen for the simulation (10^15^*cm*^−3^); therefore, the plasma-wakefield excitation has also been investigated for different values of the background plasma density up to 10^16^ *cm*^−3^ and for a fixed value of L = 105 *μ*m (Fig. 4). The parametric study performed by varying the plasma density has been conducted up to 10^16^ *cm*^−3^ to satisfy the condition *ω*_*p*_/*ω*_*THZ*_ ≪ 1 (where *ω*_*p*_ and *ω*_*THZ*_ are the plasma and THz pulse pulsation, respectively) required to have an almost dispersionless propagating electromagnetic signal in a plasma.

**Figure 5:**
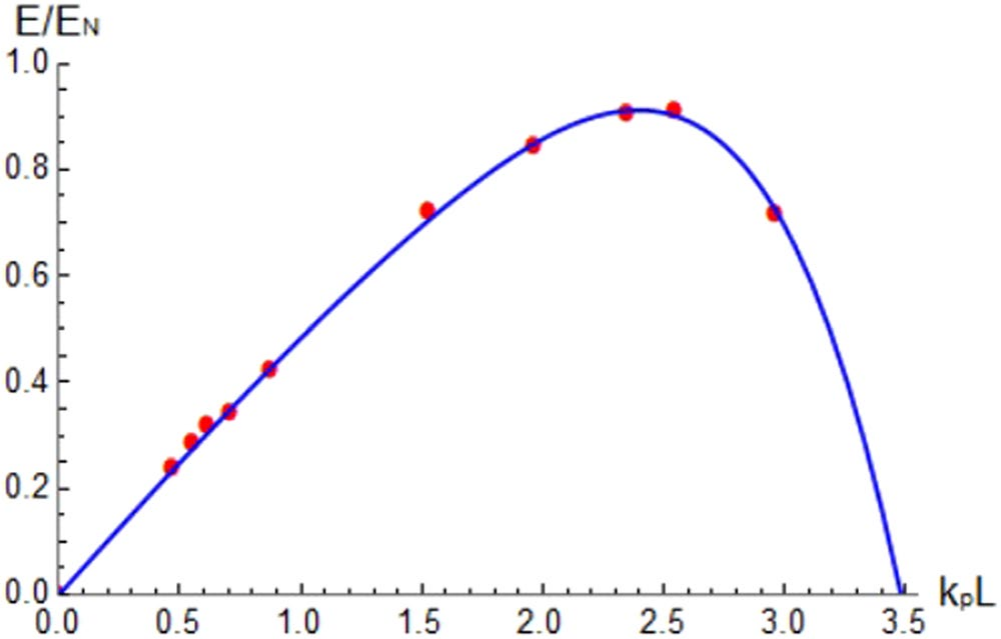
Normalized amplitude of the axial wakefield excited by single cycle THz pulses versus the parameter *k*_*p*_*L* (i.e. for any choice of background electron plasma density and THz pulse length). Normalizing amplitude: ${E}_{N}={E}_{wb}{a}_{0}^{2}\mathrm{/2}\sqrt{1+{a}_{0}^{2}\mathrm{/2}}$. Blue line: interpolation.

To provide a complete overview of the wakefield excitation process by single cycle THz pulses for any choice of background electron plasma density and THz pulse length, a 1D plot of the normalized wakefield amplitude versus the parameter *k*_*p*_*L* is reported in Fig. 5 in analogy with what reported in^33^. The amplitude values *E*_*N*_ from Eq. (2) have been used for the normalization taking into account the plasma density for each point.

The length over which THz-driven wakefields can be used to accelerate electron bunches is limited by the dephasing length:

3 $${L}_{deph}=\frac{1}{2}{{\rm{\gamma }}}_{p}^{2}{{\rm{\lambda }}}_{p}\sim \frac{1}{2}\frac{{{\rm{\lambda }}}_{p}^{3}}{{{\rm{\lambda }}}_{THz}^{2}+{{\rm{\lambda }}}_{p}^{2}{\theta }_{d}^{2}}$$

where ${{\rm{\gamma }}}_{p}=\mathrm{1/}\sqrt{1-{{\beta }}_{g}^{2}}$ is the Lorentz factor of the THz driver pulse moving inside the plasma medium with normalized group velocity ${{\beta }}_{g}=\cos \,{\theta }_{d}\sqrt{1-{{\rm{\lambda }}}_{THz}^{2}/{{\rm{\lambda }}}_{p}^{2}}$, and *θ*_*d*_ = *λ*/*π* w_0_ is the THz beam divergence. For the initial conditions given as input for the PIC code, the dephasing length is approximately 2 cm. It is possible to achieve a longer acceleration length by increasing the dephasing length. This can be done by a weaker focusing of the THz beam, by decreasing the ratio *λ*_*THz*_/*λ*_*p*_ or both. The acceleration lengths that can be achieved by exploiting the plasma wakefields can be longer than those obtained so far with capillary waveguides^11^. The *γ*_*p*_ factor, which determines the injection energy required to trap and therefore efficiently accelerate the electron bunches, sets an injection energy of a few MeV. Under the conditions assumed for our simulations, ${{\rm{\gamma }}}_{p}\simeq 6$, and the required input energy of an electron bunch is $\simeq 3$ MeV. THz-pulse-driven accelerators could boost this input energy spanning the range required for medical applications of $\simeq (1\mbox{--}20)$ MeV^41^, and therefore, they can take on an important role in this field. To better assess the potentiality of THz pulses also considering the focusing capability, a comparison with infrared pulses produced by a Ti:Sa laser is now presented and discussed. Due to its great development, the technology based on Ti:Sa has been chosen as a reference. We define the ratio *R* as

4 $$R={[\frac{{a}_{0}^{THz}}{{a}_{0}^{IR}}]}^{2}={[\frac{{{\rm{\lambda }}}_{THz}}{{{\rm{\lambda }}}_{IR}}]}^{2}\frac{{E}_{THz}}{{E}_{IR}}\frac{{{\tau }}_{IR}}{{{\tau }}_{THz}}{[\frac{{w}_{0}^{IR}}{{w}_{0}^{THz}}]}^{2}$$

where τ, *w*_0_, *E* and λ are the temporal width, the beam waist, the energy and the wavelength, respectively, for THz and IR radiation. To provide a more effective discussion with respect to the technology currently available, the following parameters have been considered.

Where z_*R*_ = $\pi {w}_{0}^{2}/{\rm{\lambda }}$ is the Rayleigh length. If the values in Table 1 are introduced in equation (4) while fixing *R* = 1, i.e., considering the same value of a_0_ both for THz and infrared, the result is

5 $$R=1\simeq 156\frac{{E}_{THz}}{{E}_{IR}}$$

where the temporal width of the IR pulse has been chosen to be equal to the temporal width of the THz pulse to excite the millimetric plasma wakefield with the same degree of resonance. If shorter IR pulses (e.g., 30 fs) are considered for a fixed energy, the driver intensity will be higher, but the pulses will be too far off the matching condition to generate significant wakefield amplitudes. On the contrary, for longer IR pulses, more energy will be required to maintain *R* = 1. On this basis (see equation (5)), one can observe that a Ti:Sa laser should deliver pulses with ∼150 times greater energy than THz pulses to excite a plasma wakefield at the same level of resonance. Therefore, from the point of view of the driver pulse energy required to excite a *λ*_*p*_ ∼ 1 mm in the quasi-non-linear regime, a THz pulse is more efficient than a Ti:Sa laser.

**Table 1:** Main characteristics of THz and IR radiation.

	λ [*μ*m]	w_0_ [*μ*m]	z_*R*_ [mm]
THz	100	200	1.3
IR	0.8	20	1.6

For example, if a plasma wave with a_0_ = 0.5 has to be excited, THz and IR energies of ${{\rm{E}}}_{THz}\simeq 10$ mJ and ${{\rm{E}}}_{IR}\simeq 1.56$ J, respectively, are required.

## Discussion and Conclusions

This paper has demonstrated that a plasma wakefield with a wavelength of 1 mm can be generated by a THz driver pulse with a central wavelength at ∼100 *μ*m. This THz pulse, as underlined in the previous sections, can currently be produced in laboratory. This excitation is found to be ∼150 times more efficient when it is triggered by the THz pulse described above rather than a Ti:Sa laser. Although, laser pulses in the optical/NIR region can currently reach energy values of tens of joules, discarding by a factor 10^4^ any THz source, from a practical point of view, the management of a laser system that delivers tens of millijoule pulses of THz radiation can be easier and cheaper than the system required to deliver the joule-level or higher level of energy required for the IR case. Moreover, the cost and complexity of a laser system grows very fast with the energy required from the system itself. Moreover, THz radiation is non-ionizing. This means that it presents fewer safety issues to the facility and simultaneously reduces the risk of damage to the equipment, thus further reducing the costs of the facility.

Despite these considerations, the objective of this work is not to replace infrared lasers in driving plasma wakefields; instead, it attempts to propose an alternative scheme that can more efficiently generate millimeter-size plasma waves that better facilitate the acceleration process as explained at the beginning of this paper.

It has to be remarked that the Ti:Sa technology is, so far, the most developed laser technology and the only technology capable of producing high peak power up to PW level at a central wavelength of approximately 800 nm. Therefore, this technology has been adopted in all experiments of laser-plasma acceleration published in the literature to date. Most of these experiments, e.g., the “3 Dream-Beams”^50–52^, have been performed in the non-linear regime, i.e., for a_0_ > 1, whereas we are interested in and therefore have investigated the quasi-non-linear regime, i.e., 0.1 < a_0_ ≤ 1.

The importance of the linear regime is underlined by the fact that there are no self-trapped electrons in the wakefield that can potentially degrade the acceleration process and the quality of the externally injected electron bunch. Therefore, the quasi-non-linear regime is the desired regime required to preserve the initial quality of the high-brightness electron bunch that has to be accelerated.

In conclusion, THz-pulse-driven plasma wakefields can be a convenient acceleration strategy that complements those relying on direct THz field acceleration. An advantage of the plasma-based THz acceleration is the linear scaling of the accelerating field with the pulse energy, in comparison with the square root behavior of direct acceleration^11–13^, THz-pulse-driven plasma wakefield acceleration can enrich the field of acceleration techniques based on plasma waves. This technique can efficiently excite high-amplitude millimetric wakefields and complements techniques based on infrared Ti:Sa and CO_2_ laser drivers^53^ (which are currently used to efficiently excite micrometer-sized wakefields). The possibility of exploiting electron plasma waves of millimetric period are expected to ease the acceleration of high-brightness externally injected electron bunches with low energy spread and high energy stability, which is particularly suitable for medical purposes and many other applications.

In addition, future developments in the area of non-linear THz-pulse-driven plasma waves (a _0_ > 1) at low electron plasma densities ∼10^15^ cm^−3^ can be promising for novel acceleration concepts, in addition to the external injection scheme discussed in this paper but for those still requiring millimetric waves and higher accelerating fields.
