# Author Correction: Resonant plasma excitation by single-cycle THz pulses

**DOI:** 10.1038/s41598-019-43377-2

**Published:** 2019-05-02

**Authors:** A. Curcio, A. Marocchino, V. Dolci, S. Lupi, M. Petrarca

**Affiliations:** 1 Department of Basic and Applied Sciences for Engineering (SBAI), “Sapienza” University of Rome, Via A. Scarpa 14, 00161 Rome, Italy; 2 Roma1-INFN, P.le Aldo Moro, 2, 00185 Rome, Italy; 3 INFN-LNF, via Enrico Fermi 40, 00044 Frascati, Italy; 4 Department of Physics, “Sapienza” University of Rome, Piazzale A. Moro 2, I-00185 Rome, Italy

Correction to: *Scientific Reports* 10.1038/s41598-017-18312-y, published online 18 January 2018

The Acknowledgements section in this Article is incomplete.

“We acknowledge financial support from the MIUR grant Rita Levi Montalcini. We acknowledge INFN-CNAF for providing the computational resources and support required for this work, and also the CINECA award under the ISCRA initiative, for the availability of high performance computing resources and support.”

should read:

“We acknowledge financial support from the MIUR grant Rita Levi Montalcini. We acknowledge INFN-CNAF for providing the computational resources and support required for this work, and also the CINECA award under the ISCRA initiative, for the availability of high performance computing resources and support. We acknowledge the contribution of the Italian Ministry of Foreign Affairs and International Cooperation, Directorate-General for the promotion of the country system for supporting the collaborative Italy-Japan project PGR00806.”

